# Circular RNAs: Novel Players in the Oxidative Stress-Mediated Pathologies, Biomarkers, and Therapeutic Targets

**DOI:** 10.1155/2021/6634601

**Published:** 2021-06-23

**Authors:** Yunguang Wang, Wenfang He, Sherif A. Ibrahim, Qiang He, Juan Jin

**Affiliations:** ^1^Department of Critical Care Medicine, Affiliated Hangzhou First People's Hospital, Zhejiang University School of Medicine, Hangzhou, Zhejiang 310006, China; ^2^Department of Nephrology, Zhejiang Provincial People's Hospital, Affiliated People's Hospital, Hangzhou Medical College, Hangzhou, Zhejiang 310014, China; ^3^Department of Histology and Cell Biology, Faculty of Medicine, Mansoura University, Mansoura, Egypt; ^4^Department of Nephrology, The First People's Hospital of Hangzhou Lin'an District, Affiliated Lin'an People's Hospital, Hangzhou Medical College, Hangzhou, Zhejiang 311300, China

## Abstract

Oxidative stress (OxS) is a wildly described cause of damage to macromolecules, resulting in abnormal physiological conditions. In recent years, a few studies have shown that oxidation/antioxidation imbalance plays a significant role in developing diseases involving different systems and organs. However, the research on the circular RNA (circRNA) roles in OxS is still in its very infancy. Therefore, we hope to provide a comprehensive overview of the recent research that explored the function of circRNAs associated with OxS and its role in the pathogenesis of different diseases that affect different body systems like the nervous system, cardiovascular system, kidneys, and lungs. It provides the possibilities of using these circRNAs as superior diagnostic and therapeutic options for OxS associated with these disease conditions.

## 1. Introduction

Circular RNA (circRNA) is a category of noncoding RNAs (ncRNAs) involved in many physiological functions and pathological processes [1]. They are ubiquitous in mammalian tissues, including humans [2–4]. They are composed of exons (ecircRNA) [5], introns (ciRNAs) [6], or a mixture of both (EIciRNAs) [7] through a process of back splicing [8]. They can be used as biomarkers to diagnose or predict some diseases [9]. In addition to their intracellular roles, circRNAs are also present in the body fluids and extracellular spaces. For example, researchers detected more than 100 circRNAs of various functional associations in human saliva [10] and thousands of different circRNA in serum and plasma [11], some of which were differentially expressed in certain diseases, including cancer, neurological diseases, and immunological disorders [12]. Those circRNAs could be either free or enclosed within exosomes [13]. Exosomes are a type of extracellular vesicles that could transfer various intracellular molecules including RNAs among cells [14]. So, circRNAs can be used as an information carrier to transmit information between and within cells, and we can use them as biomarkers for various diseases [9, 15].

Like most ncRNAs, circRNAs mostly exert their functions through the regulation of gene expression utilizing different mechanisms. The most commonly studied mechanism is the sponging of the miRNAs, hindering their functions. Thus, they represent an extra layer of gene expression regulation as they block the miRNA's inhibitory effect on protein expression. This function occurs through complementary sequences that sequester the miRNAs and prevent them from binding to the corresponding mRNAs, a process termed “sponging.” In this case, the circRNA/miRNA/mRNA axis is called the competing endogenous RNA (ceRNA). The final decision in this axis regarding the protein expression level depends on which component will have the upper hand. Recognizing these ceRNA networks and their functional roles or disease association requires a combination of computational and laboratory tools. This function is performed by ecircRNA, which resides primarily in the cytoplasm [16]. In contrast, EIcircRNA and ciRNAs are abundant in the nucleus. They enhance the expression of their parental genes of origin through the regulation of polymerase II function. They could influence either initiation or elongation of their host gene transcription [6, 17].

In addition, circRNAs could regulate gene expression by behaving like an alternative splice form of the parental gene, competing for its translatable mRNA formation. Some authors called this process an “mRNA trap” as the RNA of the gene is trapped in a circular nontranslatable form [18–20]. Alternative circularization would complement alternative splicing to form additional layer of regulation of expression of the parental genes [21]. Ma et al. showed a correlation between circRNA expression level and the expression level of their parental genes, which could be either a positive or negative correlation. This could be due to either direct regulation of parental gene expression by circRNA or due to alternative splice effect. Further investigations are mandatory to reveal the actual mechanisms [22].

Besides regulating gene expression, some circRNAs carry translatable sequences. Ribosomes could directly translate proteins from these circRNAs through internal ribosomal entry sites (IRES) or IRES-like elements [23]. Moreover, some circRNAs also directly bind the protein itself, regulating either the distribution of the protein or its activities [24, 25].

Oxidative stress (OxS) refers to the state of excess oxidative agents that exceeds the body's scavenging capacity, resulting in oxidation/antioxidation imbalance [26]. Physiologically, reactive oxygen species (ROS) regulates several vital processes like apoptosis through signal transduction and gene expression regulation. They are indispensable for some physiological functions of the body, like the destruction of invading organisms by macrophages. Under normal physiological conditions, the body's powerful antioxidant system can quickly remove excess ROS and reactive nitrogen free radicals (RNS) to maintain the dynamic balance of oxidation and antioxidation. During the normal aging process or under pathological conditions, such as hyperglycemia and stress, the antioxidant system function is disturbed, resulting in ROS or RNS accumulation, leading to oxidant-antioxidant imbalance. Alternatively, this imbalance could occur due to excessive production of these free radicals on exposure to environmental factors like smoking, pollutants, radiation, or drugs [27].

This excess of oxidants causes damage to different types of molecules, leading to gene mutation, protein denaturation, and lipid peroxidation resulting in cell senescence, apoptosis, or necrosis. Based on the affected organ, several diseases could occur. Because of the high-fat content of the nervous system, it is the most commonly affected. Neurodegenerative disorders (NDDs) like Parkinson's disease (PD) and Alzheimer's disease (AD) are examples of CNS diseases induced by OxS [28]. In the cardiovascular system (CVS), atherosclerosis and coronary artery disease are other examples [29]. OxS generates diseases of the kidney like diabetic nephropathy and acute kidney injury (AKI). OxS mediates acute lung injury of several etiologies, as well [30].

OxS is a complex process that depends on several factors and includes a wide range of metabolic pathways. So, despite the large number of available markers for the diagnosis of OxS, none of these markers is absolutely reliable by itself, which indicates the need for more accurate and definitive biomarkers for identifying the condition of OxS [26]. From a therapeutic point of view, most of the available antioxidants are nonspecific, and their effectiveness in the prevention and control of OxS associated diseases is controversial. This fact indicates the need for a more specific therapy that targets one of the pathways responsible for the regulation of OxS [27].

Like most of the vital processes, the regulation of OxS occurs on at least three levels. First, the transcriptional level in which transcription factors directly regulate the expression of the mRNA. The second level is the level of the miRNAs that inhibit the translation of the transcribed mRNAs. The circRNAs with their miRNA sponging action confer a third level of gene expression regulation. Currently, the available information about the contributions of the first and second levels in regulating or mediating the OxS ways exceeds what we know about the role of circRNA [31].

Considering the transcription factors in OxS, nuclear factor-erythroid 2-related factor 2 (Nrf2) and nuclear factor-*κ*B (NF-*κ*B) are the most important. Nrf2 is the chief transcriptional regulator of the protective action against OxS. Upon moderate stimulation, a defensive mechanism is initiated under the control of the Nrf2. Under basal conditions, Nrf2 is sequestered in the cytoplasm by the protein Keap1. Oxygen free radicals cause a conformational change of the Keap1 protein releasing Nrf2, which translocates to the nucleus. Nrf2 binds to the antioxidant response elements (ARE), inducing the expression of proteins that oppose the ROS's actions [32]. Extensive stimulation with the production of a high level of ROS switches on NF-*κ*B leading to the activation of several proteins, which could have either prooxidant or antioxidant activities in a context-dependent manner [33]. OxS also induces the forkhead box O (FoxO) protein family, which, like NF-*κ*B, could act as either a pathogenic mediator of the effect of or a protector against OxS. The mechanism responsible for directing the FoxO proteins' action in response to OxS is yet to be elucidated [34]. Other transcription factors involved in mediating the antioxidant mechanisms include activating protein-1 (AP-1) and P53. The second level of regulation is the miRNA level. Recent research discovered a long list of miRNAs that regulate the occurrence and the effects of OxS or confer antioxidant activity. They provided novel markers for diagnosis and targets for therapy. However, more effort is required to explore these miRNAs' functional roles in OxS [31]. circRNAs represent the third level of regulation. They regulate miRNA and, subsequently, mRNAs and gene expression. One advantage of circRNAs over the miRNAs is their stability, giving them better reliability as biomarkers and therapeutic agents. This stability is because the circular structure protects the circRNAs from degradation by the extracellular exonucleases [35].

The research on the circRNA roles in OxS is in its very infancy. A few recent reports have shown their actions at different levels of either protection against or mediation of the effect of OxS and involved several diseases in a few systems and organs, including the nervous system, cardiovascular system, kidney, and lung. So, this work represents a comprehensive review of all the recent research that explored the function of circRNAs associated with OxS and its role in the pathogenesis of different nonmalignant diseases that affect different body systems like the nervous system, cardiovascular system, kidneys, and lungs. It addresses the possibilities of using these circRNAs as superior diagnostic and therapeutic options for OxS associated with these disease conditions.

## 2. circRNA and OxS in the Nervous System

Both OxS and circRNAs are of particular importance in the nervous system. Brain tissue is particularly sensitive to the effect of free radicals because of the high lipid content. OxS is one of the mechanisms involved in the pathogenesis of several neurological disorders, including NDDs like glaucoma, AD, and PD [36] and intracranial infections. Despite this particular importance, previous studies failed to detect definite biological markers that would be clinically useful in diagnosing and treating these diseases and the causative OxS. The discovery of the circRNAs and their role in regulating OxS opens a new horizon in this context. circRNAs are most abundant in the CNS compared to other body systems [37], and their expression increases with the increase of age, some NDDs [38], and infections. This pattern suggests an essential role of circRNA in OxS, as it is a common factor among these conditions.

**Figure 1:**
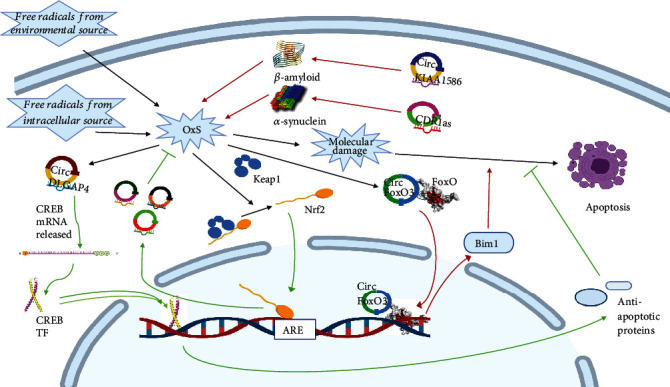
The role of circRNAs in association with OxS in neurodegenerative disorders. circRNAs play variable roles related to OxS in neurodegenerative disorders. Some of them are pathogenic (red arrows), while others are protective (green arrows). Whatever their effect is, they are pursuing different mechanisms and working at various levels to perform their functions. For example, circKIAA1586 and CDR1as mediate OxS through the sponging of miRNAs that suppress proteins that induce OxS. Others, like circFoxO3, work through binding FoxO3 protein, enhancing its nuclear entry and transcriptional activity. FoxO3 induces genes that mediate OxS-induced apoptosis. On the other hand, circDLGAP4 exerts a protective role through sponging miRNA that antagonizes CREB. CREB induces proteins that suppress the OxS-induced apoptosis. Nrf2 also has a protective effect against OxS that is mediated at least partially through circRNAs.

Few recent studies showed that a group of circRNAs is involved in OxS-mediated NDDs at different levels and roles of the regulation (Figure 1). circRNAs could be protective against, inducers of, or mediators of the effect of the OxS. From the protective side, a recent microarray examination of the circRNA differential expression in the substantia nigra and corpus striatum of Nrf2 knockout mice recognized several circRNAs that mediate the protective effect of the Nrf2/ARE pathway. Computational analysis suggested this effect to occur by regulating several ceRNA networks implicated in regulating OxS-mediated NDDs [39]. The same group did the same investigation on the hippocampus and identified several other circRNAs that could be involved. They constructed a differentially expressed ceRNA network related to the PD and studied their functional and pathway relations [40]. Another work discovered that circRNAs also play a protective role employing different pathways. One of them is circDLGAP4 which sponges and inhibits the action of miR-134-5p that typically induces oxidative stress, enhancing the progression of PD [41]. One more research on a C. elegans PD model found circzip-2 to protect against OxS through sponging another OxS enhancing miRNA, namely, miR-60-3p. The authors proposed another mechanism that still needs further verification through the competition for the expression of the zip-2 mRNA. Zip-2 protein typically enhances OxS through induction of the *α*-synuclein protein and ROS [42].

On the other side, several circRNAs contribute to the pathogenesis of NDDs through enhancing OxS or mediating its effects. This contribution again occurs through different mechanisms. For example, circFoxO3 mediates the glutamate-induced OxS and apoptosis in HT22 neuronal cells through enhancing FoxO3 protein. Silencing circFoxO3 abolished glutamate-induced ROS production and decreased FoxO3 protein expression and intranuclear translocation. Inside the nucleus, FoxO3 acts as a transcription factor that enhances the expression of the proapoptotic factor “BCL2-interacting mediator of cell death–extra long” Bim_EL_ [43]. Similarly, the expression of circRNA KIAA1586 increases in AD. circRNA KIAA1586 sponges hsa-miR-29b, inducing the accumulation of amylase-*β* [44], which, through OxS, initiates the pathogenesis of AD [45]. The circRNA KIAA1586-related ceRNA network analysis showed that it is enriched with miRNAs related to several cellular processes associated with AD. One of these processes is the perineuronal ensheathment [44], which naturally protects the neurons from the destructive effect of OxS [46]. Another circRNA of a potential role in neurodegenerative and other neurological disorders is cerebellar degeneration-related protein-1 antisense (CDR1as), which is transcribed from the CDR1 gene. It is remarkably characterized by the high number of sponge sites for the miRNA-7 [47]. miR-7 regulates several brain processes related to OxS and its associated NDDs [48]. As an example in PD, miR-7 inhibits the expression of synuclein protein posttranscriptionally [49]. As CDR1as inhibits the action of miR-7, it is expected to increase the expression of synuclein which induces OxS as part of the pathogenesis of PD [50]. Further research is required to decipher the direct role of CDR1in the regulation of OxS.

Glaucoma is another NDD that showed evidence of the role of circRNA. OxS is a causative factor of glaucoma [51]. A recent study showed that circRNA ZNF609 (cZNF609) mediates OxS-induced glaucoma-associated retinal neurodegeneration. Glaucoma rat model showed upregulation of cZNF609. The knockdown of this circRNA alleviated the pathological effects of glaucoma *in vivo* and reduced OxS-induced apoptosis of endothelial cells, retinal glial cells, and Müller cell *in vitro* and *in vivo*. On the other hand, its overexpression produced an opposite effect. These effects occur through sponging the miRNA-615-5p, releasing its inhibitory effect on the neurogenic, gliogenic, and angiogenic factor Meteorin [52].

circRNAs were also suggested to play a role in intracranial infection-mediated OxS. One example is circRNA_008636, which increased in patients with postoperative intracranial infection. Functional analysis linked circRNA_008636 to FoxO3 and AMPK pathways, which mediate OxS. So, circRNA_008636 was suggested to be a mediator of infection-induced OxS and could be used among other circRNAs as a biomarker for early diagnosis of intracranial infection [53].

## 3. circRNA and OxS in the Cardiovascular System

**Figure 2:**
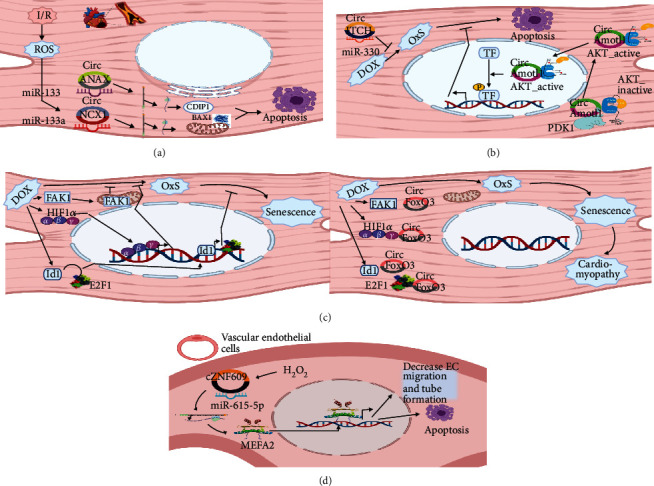
Roles of different circRNAs in different cardiovascular pathologies. (a) In myocardial infarction, both circANAX and circNCX1 mediate the effect of OxS resulting from I/R through the expression of proapoptotic proteins. (b) circAmotl1 and circITCH alleviate Dox-induced cardiomyopathy. circAmotl1 binds AKT1 and PDK1 proteins leading to AKT1 phosphorylation, activation, and nuclear translocation. Then, AKT activates transcription factors that enhance cell longevity and mitigate senescence. circITCH tethers miRNAs leading to the release of proteins that suppress the OxS. (c) circFoxO3 is involved in the pathogenesis of cardiomyopathy through capturing proteins that typically hinder this process. These proteins normally prevent OxS or counteract its effects on cell senescence. (d) Oxidative stress increases the expression of cZNF609 in vascular endothelial cells. This circRNA tethers miRNA, releasing the MEFA2 protein expression, which is a transcription factor.

OxS is one of the factors that are elemental to the pathogenesis of several cardiovascular diseases, like coronary heart disease (CAD), cardiomyopathy, and atherosclerosis (AS) (Table 1 and Figure 2). Finding novel markers of OxS associated with these conditions would be advantageous for early diagnosis and better intervention [54]. circRNA is a potential source for these markers and novel targets for treatment as they are associated with several cardiovascular disorders, including those mediated or induced by OxS [55].

Regarding coronary heart diseases, ischemia and reperfusion (I/R) induce ROS production, which consecutively induces apoptosis of cardiac muscles, followed by fibrosis [56]. Li et al. showed that H_2_O_2_ induced several members of the circRNAs in primary neonatal cardiomyocytes *in vitro*. The most abundant were circNCX1, circTLK1, and circCDYL. Among them, circNCX1 is the highest expressed, and it mediates the H_2_O_2_-induced apoptosis. This action is mediated through sponging miR-133a-3p, which generally protects the myocytes against ROS-induced apoptosis by suppressing the expression of the pro-apoptotic factor Cdinp1.

Furthermore, in the I/R mouse model, the knockdown of circNCX1 attenuates the resultant myocardial injury and improves the outcome [57]. The direct roles of circTLK1 and circCDYL are yet to be investigated. Another work by Zong and Wang showed a similar effect of circANXA2 utilizing different pathways. circANAX2, like circNCX1, showed increased expression in cardiac myoblasts after stimulation with H_2_O_2_, and it mediated the apoptotic effect. circANAX2 initiates apoptosis by sequestering miRNA-133 leading to the induction of BCL2, which is a proapoptotic protein. The miRNA-133 typically suppresses the expression of BCL2. In this work, the direct role of circANXA2 in mediating oxidative stress was tested by measuring its effect on OxS markers like superoxide dismutase (SOD) and malondialdehyde (MDA) [58]. Another circRNA, circHIPK3, regulates the talk between the different heart components to orchestrate the response to oxidative stress induced by cardiac I/R. circHIPK3 is released in exosomes from hypoxia-induced cardiomyocytes to generate an antioxidant effect on cardiac microvessel endothelial cells (CMVECs). This effect is mediated through the sponging of miR-29a, releasing the inhibitory effect on IFG1 expression. IFG1 has an antiapoptotic role [59].

Oxidative stress also mediates the pathogenesis of cardiomyopathy of different etiologies. One of the circRNAs involved in this process is circFoxO3. As occurs in the brain, circFoxO3 mediates the effect of OxS in the heart, albeit utilizing a different mechanism and a different pathway. First, circFoxO3 expression increases in the heart with aging, suggesting a possible relation to OxS. Also, *in vitro* exposure of the cardiomyocytes to H_2_O_2_ showed an increase in circFoxO3 expression. Cardiac myocyte cells utilize HIF1*α* and FAK1 transcription factors to antagonize the oxidative stress-induced senescence. circFoxO3 antagonizes the protective action of these transcription factors by decoying and trapping them in the cytoplasm, hindering their nuclear translocation. This way, it enhances the effect of oxidative stress [60]. In the opposite direction, circITCH reduces doxorubicin-induced cardiomyopathy. Doxorubicin is a chemotherapeutic drug that is cardiotoxic and causes OxS-induced cardiomyopathy by enhancing apoptosis. circITCH alleviates doxorubicin-mediated mitochondrial and cellular ROS production through sponging of the miR-330-5p. When circITCH is overexpressed in mice, it alleviated the doxorubicin-induced cardiomyopathy [61]. Another study using a mouse model of doxorubicin-induced cardiomyopathy found that circ-Amotl1 has a similar protective action. circ-Amotl1 acts as a scaffold that brings protein kinase B (AKT) and 3-phosphoinositide-dependent kinase-1 (PDK1) together, facilitating AKT phosphorylation by PDK1, leading to AKT activation and nuclear translocation [62]. AKT was proved before to attenuate the effect of H_2_O_2_ on the cardiac myocytes [63, 64] and vascular smooth muscle cells [65]. Direct testing of the relation between circ-Amotl1 and oxidative stress is still required. Both circITCH and circ-Amotl1 could be used as antioxidant therapy to avoid the side effect of such indispensable medicine.

Several predisposing factors of AS exert their effect through ROS production and, subsequently, OxS, including smoking, hypertension, hypercholesterolemia, and diabetes mellitus [66]. Furthermore, different steps of the pathogenesis of atherosclerosis are associated with the ROS release, starting with the LDL's oxidation and ending with the ROS produced by the foam cells [67]. Understanding the mechanisms of induction and mediation of the effect of OxS is axial to finding biomarkers for early diagnosis and targets for the treatment of the disease. circANARIL participates in mediating the OxS activity during the pathogenesis of AS. Overexpressing circANARIL in endothelial cells derived from the aorta of rat models of AS led to increased OxS markers such as SOD and MDA [68].

The endothelial cell (EC) dysfunction is central to several vascular diseases, including atherosclerosis and retinal vascular diseases. Again, the control of oxidative activity is vital in healthy ECs, and its imbalance leads to EC dysfunction. A study by Liu et al. showed that the circRNA ZNF609 is essential in the pathogenesis of the OxS-induced EC dysfunction. The knockdown of the cZNF609 *in vitro* and *in vivo* protected ECs against the OxS-induced apoptosis and enhanced its proliferation, migration, and tube formation. This effect occurred through sponging miR-615-5P, which naturally inhibits MEF2A. MEF2A release by the cZNF609 will induce apoptosis and decrease migration and tube formation by ECs *in vitro*. In the same study, overexpression of cZNF609 resulted in an opposite effect confirming its pathogenic role in OxS [69].

## 4. circRNA and OxS in the Kidney

**Figure 3:**
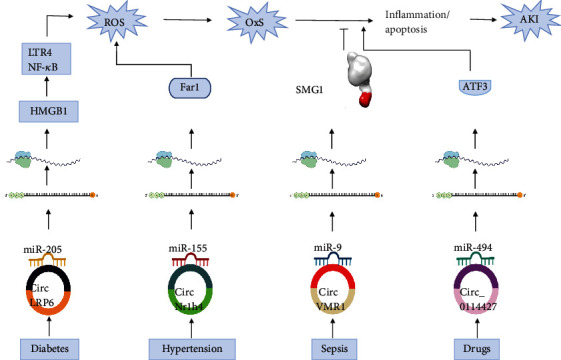
The role of circRNAs in OxS-moderated AKI. Several factors cause AKI, including diabetes, hypertension, sepsis, and drugs like cisplatin and Losartan. The pathogenesis involves the excessive production of ROS, leading to OxS that triggers inflammation and apoptosis, which finally produces AKI. circRNAs play different roles in this process with different etiologies. All of them work through tethering distinct miRNAs that release mRNA to express proteins with various actions.

Acute kidney injury (AKI) is caused by several factors, including diabetes, sepsis, drugs [70], and hypertension [71]. It is characterized by a depressed glomerular filtration rate and, subsequently, creatinine accumulation in the blood [70]. OxS is central in AKI pathogenesis regardless of the cause. Recent studies have addressed the involvement of circRNA in OxS-induced AKI (Table 2 and Figure 3), and their effect could be either mediating or protecting [30]. For example, in diabetic nephropathy (DN), circLRP6 mediates the OxS associated with mesangial cell injury due to hyperglycemia. This effect occurs through the sponging of miR-205, leading to activation of the LTR4/NF-*κ*B [72]. In this instance, NF-*κ*B acts as a promoter of the action of OxS. Similarly, circNr1h4 prompts ROS synthesis that leads to AKI in the hypertensive mouse model through sponging miR-155-5P leading to activation of fatty acid reductase 1 (Far1) with subsequent increase of the ROS leading to tissue damage [73].

Another research shows that several circRNAs are differentially expressed in the cisplatin-induced AKI (CI-AKI) mouse model. Among them, has_circRNA_0114427 was the most differentially expressed, and H_2_O_2_ induced its expression in cultured human kidney cells. These results suggest that has_circRNA_0114427 mediates the effect of oxidative stress caused by cisplatin. Further investigations showed that has_circRNA_0114427/miRNA-494-ATF3 axis is the ceRNA involved in this process [74]. Additional studies are required to identify the role of other individual circRNAs in this process, enabling us to find new markers for diagnosis and targets for CI-AKI therapy.

Contrary to this effect, circVMA21 alleviates the effect of OxS caused by sepsis. Both *in vitro* and *in vivo* experiments showed that sepsis reduces the expression of circVMA21 in kidney cells and tissues. Overexpression of circVMA21 reduced sepsis-associated oxidative damage and its subsequent apoptosis and inflammation, leading to AKI. circVMA21 exerts its effect by sponging miR-9-3P, which generally suppresses the SMG1 protein. The SMG1 protein has a protective effect through alleviating the OxS-induced inflammation and apoptosis [75]. An additional study showed that circRNAs mediated the action of Losartan in treating I/R-induced AKI. Functional analysis of these circRNAs showed that the biological process of some of them involved antioxidant activity. Pathway analysis showed that other circRNAs were related to FoxO signaling, which is also associated with OxS [76]. Again, further exploration of the individual circRNAs recognizes either markers of diagnosis or treatment of the effect of I/R-induced AKI.

## 5. circRNA and OxS in the Lung

Once more, OxS is essential in the pathogenesis of several pathological lung conditions, including fibrosis, chronic obstructive pulmonary disease (COPD), acute lung injury, and aging [77]. OxS is of particular importance in the lungs as they lie in the interface between the body and the environment, so it is more exposed to external sources of free radicals [78].

Because the study of circRNAs and their role in human pathology is a starting new field, very few papers investigated the direct role of circRNAs in OxS in the lung. Two different groups found circRNAs involved in the pathogenesis of COPD induced by cigarette smoking. Both of them found their circRNA to be differentially expressed between smokers with COPD and non-COPD individuals and in cells induced with cigarette smoke extract (CSE) *in vitro*. As the oxidative injury is central to the pathogenesis of COPD, which is mainly induced by cigarette smoking, they studied the role of these circRNAs in mediating OxS in such a condition. One group tested the role of circRNA ankyrin repeat domain (ANKRD) 11 (circANKRD11) in cigarette smoking-induced apoptosis, inflammation, and oxidative stress. The circANKRD11 works by regulating the ceRNA axis, including circRNKRD11, miR-145-5p, and the bromodomain-containing 4 (BRD4) mRNA that mediates the negative effects of cigarette smoking. They evaluated the OxS by measuring ROS, MDA, and SOD activity [79]. Similarly, the other group found circRNA oxysterol binding protein like 2 (circOSBPL2) to induce apoptosis, inflammation, and oxidative stress in response to CSE. This effect was mediated by the ceRNA axis and circOSBPL2/miR-193a-5p/BRD4 [80]. This work indicates an important role of circRNAs in OxS-mediated pathology of the lung, a phenomenon that needs further investigation to elucidate novel markers for diagnosis and targets for control of such conditions.

In addition, a few studies are indirectly suggestive of a role of circRNAs in facilitating the OxS-mediated lung injuries. For example, a research group found several circRNAs to be differentially expressed in a mouse model of silicosis. Because HECT domain-containing E3 ubiquitin-protein ligase 1 (HECTD1) mediates-silicosis associated macrophage stimulation and fibrosis, they selectively studied the role of circHECTD1 in silicosis. circHECTD1 was upregulated in the mouse model of silicosis. Their studies included the direct role of circHECTD1/HECTD1 axis in fibroblast [81] and its indirect effect through endothelial cells [82] and macrophages [83], leading to induction of lung fibrosis after stimulation with SiO. An additional study by the same group confirmed the role of circ_012091 in mediating the silicosis-induced lung fibrosis as it was downregulated in the mouse model of silicosis and after induction of lung cells with SiO *in vitro* [35]. Likewise, radon-induced lung injury was associated with the increase of several circRNAs. Because the OxS mediates the lung injury caused by radon, these circRNAs could be involved in the OxS action [84]. However, both in silicosis and radon-induced lung injury, the direct involvement of the circRNAs in OxS was not tested. Investigating this relation is mandatory as these circRNAs would be vital either as diagnostic tools of the presence of OxS or as therapeutic targets that would alleviate the effect of these harmful therapeutic or environmental factors.

## 6. And What Else?

A few more studies explored the role of circRNAs in OxS in different tissues, systems, or diseases (Table 3). Most of them tested the direct effect of OxS through induction with H_2_O_2_. One study used the circRNA-seq and bioinformatics analysis to detect the circRNAs related to pathways involved in OxS among those circRNAs that are differentially expressed in cataract patients. In this work, they recognized circZNF292 as an antioxidative factor that alleviates the effect of OxS in the eye lens, thus reducing cataract occurrence. They also validated miR-23b-3p as the downstream miRNA. Direct experiments are required to confirm the role of this network in alleviating the effect of cataract inducing OxS [85]. Liu et al. used the same approach and recognized circHIPK3 as an antioxidative factor that works through sponging miR-193a leading to activation of CRYAA. CRYAA is typically expressed in a clear lens and inhibits apoptosis. The researchers directly confirmed the role of this ceRNA as an antioxidant by testing the effect of H_2_O_2_ on the lens epithelial cells *in vitro* [86]. In an *in vitro* investigation on skin cells, cZNF609 mediated the effect of OxS through sponging miR-145 leading to induction of the JNK/P38 pathway. H_2_O_2_ induced ROS、cell damage and apoptosis through this cZNF609-related ceRNA [87]. This effect is analogous to its role in vascular endothelial cells but employing a different pathway as discussed above [69]. In parallel research on hepatic cell lines *in vitro*, circRNA4099 performed a comparable action through sponging miR-706. In addition to its destructive effect, circRNA4099 induces liver fibrosis [88]. Previous work on the same cell line showed that miR-706 mitigates the liver cell fibrosis induced by H_2_O_2_ through regulation of PKC*α* and TAOK1 signaling pathways [89].|In another study in H_2_O_2_-stimulated human dental pulp stem cells (DPSCs), sequencing of the differentially expressed circRNAs showed a long list of circRNAs. Functional and pathway analysis showed that the most prominent pathways associated with these circRNAs are P53, cell cycle, and MAPK pathways [90].

## 7. Discussion and Conclusion

Understanding the mechanisms of OxS is crucial as it helps us balance the oxidant-antioxidant status, which could be harmful if it goes extreme in either direction. Finding novel markers and therapeutic targets for OxS is mandatory for preventing and treating several diseases initiated by OxS. Most of the available antioxidants are nonspecific, and the results regarding their effectiveness in the prevention of diseases are controversial. The little research performed on the role of the circRNAs in OxS shows a promising future as they represent a third level of regulation of the process. These results show that these circRNAs could have therapeutic or diagnostic roles (see Tables 1 and 4). As a therapeutic factor, the circRNA itself could act as an antioxidant like the circDLGAP4 and circzip-2 in PD, circITCH, and Amotl1 in doxorubicin-induced cardiomyopathy and circANRIL in atherosclerosis. Other types could be novel targets for antioxidants such as circNCX1 and circANXA2 in ischemic heart disease. An example of possible diagnostic use is circRNA_008636 which could be an early diagnostic marker for intracranial infection. However, till now, no circRNA has entered clinical trials, and much more effort is needed to evaluate the efficiency of these therapeutic tools or markers in clinical settings.

As indicated in our review, some circRNAs potentially play a universal role in OxS in different organs and systems. An example is the circFoxO3, which was found by two independent studies to be associated with OxS both in the nervous system leading to NDD and in the heart protecting from the drug-induced cardiomyopathy. circFoxO3 here is an example of factors that could have different effects in different contexts. Likewise, cZNF609 was found by separate authors to play a protective role against OxS in retinal neurodegeneration [52], endothelial cell dysfunction [69], and pressure ulcer of the skin [87]. Also, circHIPK3 alleviates the OxS effect both in the heart cells and eye lens cells [59, 86]. These universal pathways would be beneficial in finding common protective measures or biomarkers for OxS. Further studies are still needed before deciding about the possibility of general or tissue-specific nature. However, studies on miRNA showed a tissue-specific nature of their action. Given the link in function between circRNA and miRNA, it is expected to find the exact nature in the former. Understanding this tissue specificity is crucial for tailoring individualized therapy for each condition.

Additional investigations are mandatory to fully understand the role of the discovered circRNAs and find other novel ones that would help us understand the networks that control and mediate the effect of OxS. All of the previous studies started with finding the circRNA then linking it to the miRNA or the protein in order to understand its function. As we already have a list of proteins and miRNA associated with OxS [31], we would suggest a reverse approach that starts with the proteins and miRNAs. For protein-related studies, protein immunoprecipitation assay followed by circRNA-seq could be pursued [24]. For the miRNA, computational predictions followed by confirmation with RNA pull-down assay could be utilized [91]. Previous information available from miRNA and lncRNA opens the door for getting promising results from the circRNAs. However, circRNAs would be adventitious as they show more stability in the extracellular space.

## Figures and Tables

**Table 1:** The effect of circRNAs on OxS in the cardiovascular system.

circRNA	Mediators	Effect	Disease	Model	Clinical application	Ref
circNCX1	miR-133a-3p	Mediates the ROS-induced apoptosis in ischemic myocardial injury	Ischemic heart disease	H9c2 cell line (H_2_O_2_ treated), neonatal cardiomyocytes and mouse model of myocardial infarction	Target for antioxidant therapy	[57]
circANXA2	miR-133 BCL2	Same as circNCX1	Ischemic heart disease	H9c2 cell line (H_2_O_2_ treated)	Target for antioxidant therapy	[58]
circHIPK3	miR-29a and IFG1	Suppresses the OxS-induced apoptosis	Ischemic heart disease	CMVECs	Antioxidant therapy	[59]
circFoxO3	Antistress proteins FAK and HIF1a	Mediates oxidative stress-induced senescence	Cardiomyopathy	Cell culture and mouse model	Target for antioxidant therapy	[60]
circITCH	miR-330-5p	Reduces the mitochondrial and cellular ROS production	Doxorubicin-induced cardiomyopathy	Cell culture, mouse model	Antioxidant therapy	[61]
Amotl1	AKT and PKD1 proteins	Mediates the activity of OxS	Doxorubicin-induced cardiomyopathy	Cell culture, mouse model	Antioxidant therapy	[62]
circANRIL	NA	Inhibits SOD activity and reduces atherosclerosis-associated cell damage	Atherosclerosis	*In vitro* EC derived from Aorat of an atherosclerotic rat model	Antioxidant therapy	[68]
circRNA ZNF609	miR-615-5p-MEF2A	Reduces OxS-associated retinal vessel loss and pathological angiogenesis	Vascular endothelial dysfunction	HUVRCs OIR mouse model	Target for antioxidant therapy	[69]

NCX1: sodium/calcium exchanger 1; circANRIL: circular antisense noncoding RNA in the INK4 locus; PDK: phosphoinositide-dependent kinase.

**Table 2:** The role of circRNAs in OxS in kidney diseases.

Cause	circRNA	Mediator	Mechanism	Role	Ref
Diabetes	circLRP6	miR-205 LTR4/NF-*κ*B	Mediates the effect of ROS on mesangial cells	Pathogenic	[72]
Hypertension	circNr1h4	miR-155-5P Far1	Prompts ROS synthesis	Pathogenic	[73]
Drug-induced	circ_0114427	miR-494 ATF3	Mediates the effect of ROS	Pathogenic	[74]
Sepsis	circVMA21	miR-9-3P SMG1 protein	Alleviates OxS mediated inflammation and apoptosis	Protective	[75]

**Table 3:** The study of circRNAs on OxS in different tissues, systems, or diseases.

Tissue	circRNA	Mediator	Mechanism	Clinical application	Study method	Ref
Ocular: cataract	circZNF292	miR-23b-3p	Alleviates the effect of OxS	Antioxidative therapy	RNA-seq and computational analysis	[85]
Ocular: cataract	circHIPK3	miR-193a/CRYAA	Alleviates the effect of OxS	Antioxidative therapy	RNA-seq and computational analysis. Confirmed by the effect of H_2_O_2_*in vitro*	[86]
Skin	circZNF609	miR-145 -JNK/P38 pathway	Mediates the effect of OxS	Target for antioxidative therapy	Effect of H_2_O_2_*in vitro*	[87]
Liver	circRNA_4099	miR-706	Mediates the H_2_O_2_-induced apoptosis	Target for antioxidative therapy	Effect of H_2_O_2_ on hepatic cells *in vitro*	[88]
Dental	Multiple	Multiple	N/A	N/A	Effect of H_2_O_2_*in vitro*	[90]

**Table 4:** The potential for the use of circRNAs as diagnostic and therapeutic tools for OxS-induced nervous system diseases.

circRNA	Mediators	Effect	Disease	Model	Clinical application	Ref
mmu_circRNA_34132 mmu_circRNA_017077 mmu_circRNA_015216	miR-27 and miR-34a	Mediates the protective effect of Nrf2	PD	Substantia nigra and corpus striatum of Nrf2 (-/-) mice and in silico	Antioxidant therapy	[39]
circDLGAP4	miR-134-5p/CREB	Reduces OxS	PD	SH-SY5Y and MN9D cell lines and mouse model	Biomarker and antioxidative therapy	[41]
circzip-2	Zip2 miR-60-3p *α*-synuclein Daf-16 pathway	Reduces ROS production	PD	Transgenic C. elegans model of PD	Biomarker and antioxidative therapy	[42]
circFoxO3	FoxO3-Bim_EL_ pathway	Mediates glutamate-induced OxS	NDD	*In vitro* neuronal cell culture (HT22 cells)	Target for therapy	[43]
circRNA KIAA1586	hsa-miR-29b	Induces amyloid *β* protein	AD	In silico	Biomarker and therapeutic target	[44, 45]
CDR-1as	miR-7 *α*-synuclein protein	Mediates the initiation of OxS (suggested)	PD	Several studies	Target for therapy	[50, 54]
circRNA ZNF609	miR-615-metrorin (METRN) protein	Mediates the response to OxS	Retinal NDD	Muller cells and a rat model of retinopathy	Target for therapy	[52]
circRNA_008636	FoxO3 AMPK pathways	Mediates infection-induced OxS	CNS infection	Postoperative patients	A diagnostic marker for infection	[53]

## Data Availability

The data used to support the findings of this study are included within the article.

## References

[1] Kristensen L. S., Andersen M. S., Stagsted L. V. W., Ebbesen K. K., Hansen T. B., Kjems J. (2019). The biogenesis, biology and characterization of circular RNAs. *Nature Reviews Genetics*.

[2] Xu T., Wu J., Han P., Zhao Z., Song X. (2017). Circular RNA expression profiles and features in human tissues: a study using RNA-seq data. *BMC genomics*.

[3] Chen L., Huang C., Wang X., Shan G. (2015). Circular RNAs in eukaryotic cells. *Current Genomics*.

[4] Holdt L. M., Kohlmaier A., Teupser D. (2018). Molecular roles and function of circular RNAs in eukaryotic cells. *Cellular and Molecular Life Sciences*.

[5] Chen I., Chen C. Y., Chuang T. J. (2015). Biogenesis, identification, and function of exonic circular RNAs. *Wiley Interdisciplinary Reviews: RNA*.

[6] Zhang Y., Zhang X. O., Chen T. (2013). Circular intronic long noncoding RNAs. *Molecular Cell*.

[7] Hu Q., Zhou T. (2018). EiciRNA-mediated gene expression: tunability and bimodality. *FEBS Letters*.

[8] Li X., Yang L., Chen L. L. (2018). The biogenesis, functions, and challenges of circular RNAs. *Molecular Cell*.

[9] Abu N., Jamal R. (2016). Circular RNAs as promising biomarkers: a mini-review. *Frontiers in Physiology*.

[10] Bahn J. H., Zhang Q., Li F. (2015). The landscape of microRNA, piwi-interacting RNA, and circular RNA in human saliva. *Clinical Chemistry*.

[11] Vea A., Llorente-Cortes V., de Gonzalo-Calvo D. (2018). Circular RNAs in blood. *Advances in Experimental Medicine and Biology*.

[12] Chen Y., Li C., Tan C., Liu X. (2016). Circular RNAs: a new frontier in the study of human diseases. *Journal of Medical Genetics*.

[13] Fanale D., Taverna S., Russo A., Bazan V. (2018). Circular RNA in exosomes. *Advances in Experimental Medicine and Biology*.

[14] Ibrahim S. A., Khan Y. S. (2020). *Histology, extracellular vesicles*.

[15] Ren S., Lin P., Wang J. (2020). Circular RNAs: promising molecular biomarkers of human aging-related diseases via functioning as an miRNA sponge. *Molecular Therapy Methods & Clinical Development*.

[16] Rong D., Sun H., Li Z. (2017). An emerging function of circRNA-miRNAs-mRNA axis in human diseases. *Oncotarget*.

[17] Li Z., Huang C., Bao C. (2015). Exon-intron circular RNAs regulate transcription in the nucleus. *Nature Structural & Molecular Biology*.

[18] Jeck W. R., Sharpless N. E. (2014). Detecting and characterizing circular RNAs. *Nature Biotechnology*.

[19] Chao C. W., Chan D. C., Kuo A., Leder P. (1998). The mouse formin (Fmn) gene: abundant circular RNA transcripts and gene-targeted deletion analysis. *Molecular Medicine*.

[20] Jeck W. R., Sorrentino J. A., Wang K. (2013). Circular RNAs are abundant, conserved, and associated with ALU repeats. *RNA*.

[21] Zhang X. O., Wang H. B., Zhang Y., Lu X., Chen L. L., Yang L. (2014). Complementary sequence-mediated exon circularization. *Cell*.

[22] Ma N., Tie C., Yu B., Zhang W., Wan J. (2020). Circular RNAs regulate its parental genes transcription in the AD mouse model using two methods of library construction. *FASEB Journal*.

[23] Mo D., Li X., Raabe C. A. (2019). A universal approach to investigate circRNA protein coding function. *Scientific Reports*.

[24] Huang A., Zheng H., Wu Z., Chen M., Huang Y. (2020). Circular RNA-protein interactions: functions, mechanisms, and identification. *Theranostics*.

[25] du W. W., Zhang C., Yang W., Yong T., Awan F. M., Yang B. B. (2017). Identifying and characterizing circRNA-protein interaction. *Theranostics*.

[26] Sies H. (2015). Oxidative stress: a concept in redox biology and medicine. *Redox Biology*.

[27] Vassalle C., Maltinti M., Sabatino L. (2020). Targeting oxidative stress for disease prevention and therapy: where do we stand, and where do we go from here. *Molecules*.

[28] Salim S. (2017). Oxidative stress and the central nervous system. *The Journal of Pharmacology and Experimental Therapeutics*.

[29] Dubois-Deruy E., Peugnet V., Turkieh A., Pinet F. (2020). Oxidative stress in cardiovascular diseases. *Antioxidants*.

[30] Ozbek E. (2012). Induction of oxidative stress in kidney. *International Journal of Nephrology*.

[31] Engedal N., Žerovnik E., Rudov A. (2018). From oxidative stress damage to pathways, networks, and autophagy via microRNAs. *Oxidative Medicine and Cellular Longevity*.

[32] Bellezza I., Giambanco I., Minelli A., Donato R. (2018). Nrf2-keap1 signaling in oxidative and reductive stress. *Biochimica et Biophysica Acta.Molecular Cell Research*.

[33] Lingappan K. (2018). NF-*κ*B in oxidative stress. *Current Opinion in Toxicology*.

[34] Storz P. (2011). Forkhead homeobox type o transcription factors in the responses to oxidative stress. *Antioxidants & Redox Signaling*.

[35] Cheng Y., Luo W., Li Z. (2019). CircRNA_012091/PPP1R13B-mediated lung fibrotic response in silicosis via endoplasmic reticulum stress and autophagy. *American Journal of Respiratory Cell and Molecular Biology*.

[36] Jia E., Zhou Y., Liu Z. (2020). Transcriptomic profiling of circular RNA in different brain regions of Parkinson's disease in a mouse model. *International Journal of Molecular Sciences*.

[37] You X., Vlatkovic I., Babic A. (2015). Neural circular RNAs are derived from synaptic genes and regulated by development and plasticity. *Nature Neuroscience*.

[38] Gruner H., Cortés-López M., Cooper D. A., Bauer M., Miura P. (2016). CircRNA accumulation in the aging mouse brain. *Scientific Reports*.

[39] Yang J. H., Zhang R. J., Lin J. J. (2018). The differentially expressed circular RNAs in the substantia nigra and corpus striatum of Nrf2-knockout mice. *Cellular Physiology and Biochemistry : International Journal of Experimental Cellular Physiology, Biochemistry, and Pharmacology*.

[40] Zhang R. J., Li Y., Liu Q. (2019). Differential expression profiles and functional prediction of circular RNAs and long non-coding RNAs in the hippocampus of Nrf2-knockout mice. *Frontiers in Molecular Neuroscience*.

[41] Feng Z., Zhang L., Wang S., Hong Q. (2020). Circular RNA circDLGAP4 exerts neuroprotective effects via modulating miR-134-5p/CREB pathway in Parkinson's disease. *Biochemical and Biophysical Research Communications*.

[42] Kumar L., Shamsuzzama, Jadiya P., Haque R., Shukla S., Nazir A. (2018). Functional characterization of novel circular RNA molecule, circzip-2 and its synthesizing gene zip-2 in C. elegans model of Parkinson's disease. *Molecular Neurobiology*.

[43] Lin S. P., Hu J., Wei J. X. (2020). Silencing of circFoxO3 protects HT22 cells from glutamate-induced oxidative injury via regulating the mitochondrial apoptosis pathway. *Cellular and Molecular Neurobiology*.

[44] Zhang Y., Yu F., Bao S., Sun J. (2019). Systematic characterization of circular RNA-associated ceRNA network identified novel circRNA biomarkers in Alzheimer's disease. *Frontiers in Bioengineering and Biotechnology*.

[45] Butterfield D. A., Swomley A. M., Sultana R. (2013). Amyloid*β*-peptide (1-42)-induced oxidative stress in Alzheimer disease: importance in disease pathogenesis and progression. *Antioxidants & Redox Signaling*.

[46] Cabungcal J. H., Steullet P., Morishita H. (2013). Perineuronal nets protect fast-spiking interneurons against oxidative stress. *Proceedings of the National Academy of Sciences of the United States of America*.

[47] Hansen T. B., Jensen T. I., Clausen B. H. (2013). Natural RNA circles function as efficient microRNA sponges. *Nature*.

[48] Shao Y., Chen Y. (2016). Roles of circular RNAs in neurologic disease. *Frontiers in Molecular Neuroscience*.

[49] Doxakis E. (2010). Post-transcriptional regulation of alpha-synuclein expression by mir-7 and mir-153. *Journal of Chemical Biology*.

[50] Junn E., Lee K. W., Jeong B. S., Chan T. W., Im J. Y., Mouradian M. M. (2009). Repression of alpha-synuclein expression and toxicity by microRNA-7. *Proceedings of the National Academy of Sciences of the United States of America*.

[51] Weinreb R. N., Aung T., Medeiros F. A. (2014). The pathophysiology and treatment of glaucoma: a review. *JAMA*.

[52] Wang J. J., Liu C., Shan K. (2018). Circular RNA ZNF609 regulates retinal neurodegeneration by acting as miR-615 sponge. *Theranostics*.

[53] Niu J., Xu H., Yang B. (2020). Intracranial infections in neurological surgery: the changes of circular RNA expression and their possible function mechanism. *BioMed Research International*.

[54] Dong Y., Xu W., Liu C., Liu P., Li P., Wang K. (2019). Reactive oxygen species related noncoding RNAs as regulators of cardiovascular diseases. *International Journal of Biological Sciences*.

[55] Bei Y., Yang T., Wang L. (2018). Circular RNAs as potential theranostics in the cardiovascular system. *Molecular Therapy Nucleic Acids*.

[56] Granger D. N., Kvietys P. R. (2015). Reperfusion injury and reactive oxygen species: the evolution of a concept. *Redox Biology*.

[57] Li M., Ding W., Tariq M. A. (2018). A circular transcript of ncx1gene mediates ischemic myocardial injury by targeting miR-133a-3p. *Theranostics*.

[58] Zong L., Wang W. (2020). CircANXA2 promotes myocardial apoptosis in myocardial ischemia-reperfusion injury via inhibiting miRNA-133 expression. *International Journal of Biomedical Research*.

[59] Wang Y., Zhao R., Liu W. (2019). Exosomal circHIPK3 released from hypoxia-pretreated cardiomyocytes regulates oxidative damage in cardiac microvascular endothelial cells via the miR-29a/IGF-1 pathway. *Oxidative Medicine and Cellular Longevity*.

[60] du W. W., Yang W., Chen Y. (2016). FoxO3 circular RNA promotes cardiac senescence by modulating multiple factors associated with stress and senescence responses. *European Heart Journal*.

[61] Han D., Wang Y., Wang Y. (2020). The tumor-suppressive human circular RNA circITCH sponges miR-330-5p to ameliorate doxorubicin-induced cardiotoxicity through upregulating SIRT6, survivin, and SERCA2a. *Circulation Research*.

[62] Zeng Y., du W. W., Wu Y. (2017). A circular RNA binds to and activates AKT phosphorylation and nuclear localization reducing apoptosis and enhancing cardiac repair. *Theranostics*.

[63] Lv G., Shao S., Dong H., Bian X., Yang X., Dong S. (2014). MicroRNA-214 protects cardiac myocytes against H_2_O_2_-induced injury. *Journal of Cellular Biochemistry*.

[64] Chen T., Ding G., Jin Z., Wagner M. B., Yuan Z. (2012). Insulin ameliorates miR-1-induced injury in H9c2 cells under oxidative stress via Akt activation. *Molecular and Cellular Biochemistry*.

[65] Peng J., He X., Zhang L., Liu P. (2018). MicroRNA‑26a protects vascular smooth muscle cells against H_2_O_2_‑induced injury through activation of the PTEN/AKT/mTOR pathway. *International Journal of Molecular Medicine*.

[66] Niemann B., Rohrbach S., Miller M. R., Newby D. E., Fuster V., Kovacic J. C. (2017). Oxidative stress and cardiovascular risk: obesity, diabetes, smoking, and pollution: part 3 of a 3-part series. *Journal of the American College of Cardiology*.

[67] Marchio P., Guerra-Ojeda S., Vila J. M., Aldasoro M., Victor V. M., Mauricio M. D. (2019). Targeting early atherosclerosis: a focus on oxidative stress and inflammation. *Oxidative Medicine and Cellular Longevity*.

[68] Shi P., Ji H., Zhang H., Yang J., Guo R., Wang J. (2020). circANRIL reduces vascular endothelial injury, oxidative stress and inflammation in rats with coronary atherosclerosis. *Experimental and Therapeutic Medicine*.

[69] Liu C., Yao M. D., Li C. P. (2017). Silencing of circular RNA ZNF609 ameliorates vascular endothelial dysfunction. *Theranostics*.

[70] Basile D. P., Anderson M. D., Sutton T. A. (2012). Pathophysiology of acute kidney injury. *Comprehensive Physiology*.

[71] James M. T., Grams M. E., Woodward M. (2015). A meta-analysis of the association of estimated GFR, albuminuria, diabetes mellitus, and hypertension with acute kidney injury. *American Journal of Kidney Diseases*.

[72] Chen B., Li Y., Liu Y., Xu Z. (2019). CircLRP6 regulates high glucose-induced proliferation, oxidative stress, ECM accumulation, and inflammation in mesangial cells. *Journal of Cellular Physiology*.

[73] Lu C., Chen B., Chen C. (2020). CircNr1h4 regulates the pathological process of renal injury in salt-sensitive hypertensive mice by targeting miR-155-5p. *Journal of Cellular and Molecular Medicine*.

[74] Cao Y., Mi X., Zhang D., Wang Z., Zuo Y., Tang W. (2020). Transcriptome sequencing of circular RNA reveals a novel circular RNA-has_circ_0114427 in the regulation of inflammation in acute kidney injury. *Clinical Science*.

[75] Shi Y., Sun C. F., Ge W. H., du Y. P., Hu N. B. (2020). Circular RNA VMA21 ameliorates sepsis-associated acute kidney injury by regulating miR-9-3p/SMG1/inflammation axis and oxidative stress. *Journal of Cellular and Molecular Medicine*.

[76] Fang M., Liu S., Zhou Y. (2019). Circular RNA involved in the protective effect of losartan on ischemia and reperfusion induced acute kidney injury in rat model. *American Journal of Translational Research*.

[77] Hecker L. (2018). Mechanisms and consequences of oxidative stress in lung disease: therapeutic implications for an aging populace. *American Journal of Physiology-Lung Cellular and Molecular Physiology*.

[78] Thimmulappa R. K., Chattopadhyay I., Rajasekaran S. (2019). Oxidative stress mechanisms in the pathogenesis of environmental lung diseases. *Oxidative Stress in Lung Diseases*.

[79] Wang Z., Zuo Y., Gao Z. (2021). CircANKRD11 knockdown protects HPMECs from cigarette smoke extract-induced injury by regulating miR-145-5p/BRD4 axis. *International Journal of Chronic Obstructive Pulmonary Disease*.

[80] Zheng C., Zhang Y., Zhao Y., Duan Y., Mu Q., Wang X. (2021). CircOSBPL2 contributes to smoke-related chronic obstructive pulmonary disease by targeting miR-193a-5p/BRD4 axis. *International Journal of Chronic Obstructive Pulmonary Disease*.

[81] Chu H., Wang W., Luo W. (2019). CircHECTD1 mediates pulmonary fibroblast activation vi a HECTD1. *Therapeutic Advances in Chronic Disease*.

[82] Fang S., Guo H., Cheng Y. (2018). CircHECTD1 promotes the silica-induced pulmonary endothelial-mesenchymal transition via HECTD1. *Cell Death & Disease*.

[83] Zhou Z., Jiang R., Yang X. (2018). CircRNA mediates silica-induced macrophage activation via HECTD1/ZC3H12A-dependent ubiquitination. *Theranostics*.

[84] Pei W., Tao L., Zhang L. W. (2017). Circular RNA profiles in mouse lung tissue induced by radon. *Environmental Health and Preventive Medicine*.

[85] Liang S., Dou S., Li W., Huang Y. (2020). Profiling of circular RNAs in age-related cataract reveals circZNF292 as an antioxidant by sponging miR-23b-3p. *Aging*.

[86] Liu X., Liu B., Zhou M. (2018). Circular RNA HIPK3 regulates human lens epithelial cells proliferation and apoptosis by targeting the miR-193a/CRYAA axis. *Biochemical and Biophysical Research Communications*.

[87] Ge R., Gao G. (2020). Anti-antioxidant impacts of circZNF609 silence in HaCaT cells through regulating miR-145. *Artificial Cells, Nanomedicine, and Biotechnology*.

[88] Li Y., Gao X., Wang Z. (2020). Circular RNA 4099 aggravates hydrogen peroxide-induced injury by down- regulating microRNA-706 in L02 cells. *Life Sciences*.

[89] Yin R., Guo D., Zhang S., Zhang X. (2016). miR-706 inhibits the oxidative stress-induced activation of PKC*α*/TAOK1 in liver fibrogenesis. *Scientific Reports*.

[90] Zhang J., Li D., Wang D., Man K., Yang X. (2019). CircRNA expression profiles in human dental pulp stromal cells undergoing oxidative stress. *Journal of Translational Medicine*.

[91] Jakobi T., Dieterich C. (2019). Computational approaches for circular RNA analysis. *Wiley Interdisciplinary Reviews: RNA*.

